# In-game monitoring of adolescent handball players: a preliminary examination of associations between external load parameters and objective and subjective fatigue markers

**DOI:** 10.3389/fspor.2026.1765225

**Published:** 2026-03-11

**Authors:** Julian Bauer, Thomas Muehlbauer, Jan Venzke, Robin Schäfer, Petra Platen, Markus Gruber

**Affiliations:** 1 Department of Sport Science, Human Performance Research Centre, University of Konstanz, Konstanz, Germany; 2 Division of Movement and Training Sciences/Biomechanics of Sport, University of Duisburg-Essen, Essen, Germany; 3 Department of Sports Medicine and Sports Nutrition, Ruhr University Bochum, Bochum, Germany

**Keywords:** athlete self-report measures, competition, exertion, exhaustion, team sports

## Abstract

**Background:**

Advances in local positioning systems (LPS) have enabled detailed monitoring of external load in handball. However, the relationship between external load and internal fatigue under real match conditions remains insufficiently understood, particularly in adolescent players. This study examined associations between external load parameters and both objective and subjective fatigue markers during an official match and evaluated agreement between players ‘ratings of perceived exertion (RPE) and coaches’ ratings of observed exertion (ROE).

**Methods:**

Highly trained adolescent male handball players (*n* = 11; 8 complete cases; age: 16.1 ± 0.4 years) were monitored during a Bundesliga-level youth match. External load measures included playing time, total distance covered, accelerations, decelerations, fast running, and metabolic power recorded via the Kinexon LPS. Objective fatigue was assessed using the leg recovery test (LRT), while subjective measures included the Perceived Recovery Status Scale (PRSS), RPE, and ROE. Assessments were conducted pre-game (T_0_), half-time (T_1_), and post-game (T_2_).

**Results:**

Subjective fatigue measures changed markedly across the match. RPE increased (T_0_: 8.0 ± 2.45; T_1_: 14.6 ± 2.20; T_2_: 16.1 ± 2.85), while PRSS decreased (T_0_: 8.25 ± 1.91; T_1_: 6.12 ± 1.89; T_2_: 4.75 ± 2.43). PRSS and RPE showed moderate-to-strong associations with both volume- and intensity-based external load measures after each half. In contrast, LRT scores remained largely unchanged and showed minimal associations. Agreement between RPE and ROE improved over the match (correlations: T_1_ = 0.58/0.43; T_2_ = 0.68/0.60).

**Conclusion:**

Subjective fatigue measures, particularly RPE, were more sensitive than objective neuromuscular testing for detecting match-related fatigue in adolescent handball players. Coaches’ ratings aligned increasingly with players’ perceptions, highlighting the practical value of subjective monitoring for in-game load management.

## 1 Introduction

The monitoring of athletes and their performance is a common practice in elite sports and is increasingly applied in amateur contexts, with the aim of optimising adaptation processes and reducing injury risk (1). A substantial body of monitoring literature focuses on training load, seeking to quantify the amount of load imposed on athletes within individual training sessions or across extended periods to guide training prescription and support long-term development. In this context, training-load monitoring is widely recognised as a key component of performance optimisation and long-term athlete development (2). While training-load monitoring is essential for managing adaptation processes, competition represents the context in which training outcomes are expressed and evaluated. Performance realisation during competition is therefore a key reference point for evaluating the effectiveness of training and load-management strategies (3). Nevertheless, monitoring research remains heavily skewed toward training settings, whereas the specific demands of competitive match play have received comparatively less attention.

In handball, the limitations of training-based monitoring are particularly pronounced. Competitive match play is characterised by frequent substitutions, highly variable playing time, and rapidly changing tactical and situational demands that cannot be adequately replicated in training environments. As a result, findings derived exclusively from training-based monitoring may not accurately reflect the external load and fatigue responses experienced during actual match play. This imbalance is also reflected in conceptual monitoring frameworks. For example, the updated athlete-monitoring cycle proposed by Gabbett and Oetter (4) primarily emphasises the monitoring of external and internal training loads to inform training-related decision-making, while competition load and performance realisation are only implicitly considered. Such a distinction between training- and competition-based monitoring becomes particularly relevant in team sports including handball, where match demands are highly intermittent, position-specific, and strongly shaped by tactical and situational factors, resulting in substantial inter-individual variability in external and internal load (5). Although monitoring research is well established in team sports most notably soccer [e.g., (6, 7)], evidence derived from integrated in-game monitoring approaches in handball remains limited.

Previous handball research has primarily focused on describing external match demands rather than implementing comprehensive monitoring approaches (8). Although handball has a high participation rate and considerable sporting relevance, particularly in Europe (9), the number of monitoring studies remains comparatively low. Moreover, a substantial proportion of existing research is confined to world-class or professional settings. Research in handball—especially that derived from elite sport—has largely focused either on training load or on physical performance demands quantified by objective parameters such as total distance covered (TDC) or the frequency and magnitude of accelerations and decelerations, commonly referred to as external load. Within this context, a distinction is typically made between volume-based (e.g., playing time, TDC) and intensity-based (e.g., accelerations, decelerations, fast running, metabolic power) load parameters.

One methodological advancement in the assessment of external load is the use of local positioning systems, which utilise local transmitters installed within the indoor environment to record players’ positions with high spatial resolution (10). One such system is the Kinexon local positioning system (Kinexon, Munich, Germany), which operates using ultra-wideband technology. Players wear an inertial measurement unit positioned between the scapulae in an athlete-worn sports bra (11). The device continuously transmits positional and movement data to external receivers, where the data are subsequently processed and stored (12). The Kinexon system can assess the same movement and performance variables as global positioning system (GPS) and has demonstrated high concurrent validity for handball-specific applications. Validation studies have reported absolute position errors of 8–9 cm, distance deviations of 0.6–1.0%, and speed deviations of 0.7–1.7% compared with camera-based reference systems (12). In addition, strong correlations (*r* > 0.90) and small-to-moderate typical errors of estimate (TEE: 0.06–0.48) have been reported for peak velocity and acceleration measures (10), alongside lower typical errors of estimate for distance measures compared with GPS (TEE: 1.0–6.0% (13).

Despite the detailed quantification of external load, athletes’ psychophysiological responses to these external stimuli—commonly referred to as internal load—can vary substantially between individuals. This variability may be particularly pronounced in adolescent handball, which represents a preparatory phase for the transition to higher physical demands in adult competition and is characterised by ongoing biological maturation (14) as well as heterogeneous training and match exposure histories (15). A conceptual framework that explicitly addresses internal load is the updated model of fatigue and human performance proposed by Behrens et al. (16), which differentiates between *motor performance fatigue*, *perceived motor fatigue*, *cognitive performance fatigue*, and *perceived cognitive fatigue*. One test to assess motor performance fatigue is the leg recovery test (LRT), which is based on the countermovement jump (CMJ) and is integrated into the Polar Vantage V2 sports watch (17). The LRT has been validated against force plate measurements, demonstrating a mean absolute percentage error of approximately 5%, a high correlation with force plate–derived jump height (*r* = 0.96), and no systematic bias (17). The CMJ-based LRT can be performed without imposing substantial additional load and is location-independent, making it suitable for in-game assessment contexts. In addition to objective performance-based measures, athlete self-report measures, such as questionnaires, are commonly used to assess perceived fatigue (18, 19). These measures rely on athletes’ subjective evaluations and provide rapid and easily accessible information on perceived fatigue states across different domains.

Beyond the athlete's own perspective, the assessment of internal load can be complemented by the external perspective of the coach. However, research comparing players’ perceived exertion with coaches’ ratings of observed exertion (ROE) in handball remains scarce, particularly in competitive match settings. This gap is noteworthy, as coaches’ tactical and substitution decisions during matches are largely based on visual and behavioural cues indicative of fatigue (20). Discrepancies between players’ ratings of perceived exertion (RPE) and coaches’ ROE may result in suboptimal substitution strategies and maladaptive load management over time (21). While moderate-to-high agreement between ROE and RPE has been reported in training contexts (20, 22, 23), it remains unclear whether similar levels of agreement are present during competitive match play in adolescent handball, where squad sizes are large and competitive experience is still developing. To our knowledge, this is the first in-game study in adolescent handball players to simultaneously examine associations between LPS-derived external load parameters, objective neuromuscular fatigue, subjective fatigue measures, and coach-based exertion ratings during an official match.

Accordingly, the present study addressed the following research questions: (1) Are external volume-based (playing time, TDC) and intensity-based load indicators (accelerations, decelerations, fast running, metabolic power) associated with objective (leg recovery test) and subjective (Perceived Recovery Status Scale, RPE) measures of fatigue in adolescent male handball players the end of the first and second half of the game? and (2) Are coaches’ ROE associated with players’ RPE, and do these associations differ between the end of the first and second half of the game?

## 2 Methods

### 2.1 Study design

**Figure 1:**
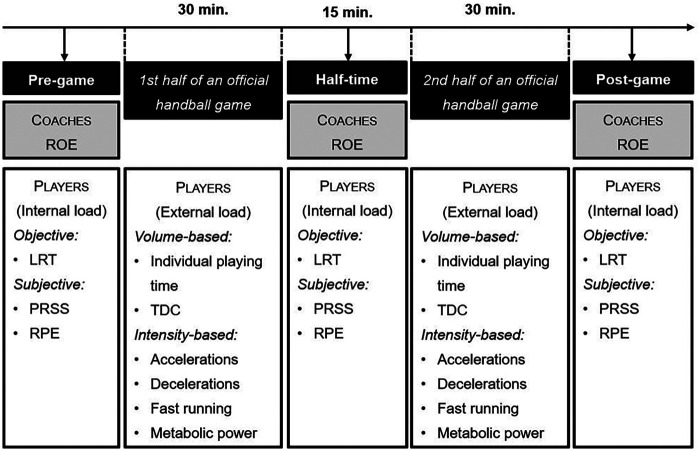
Study design of the in-game testings. LRT, leg recovery test; PRSS, Perceived Recovery Status Scale; RPE, players' rating of perceived exertion; ROE, coaches’ rating of observed exertion; TDC, total distance covered.

The study was conducted during a regular handball championship game in the b-juniors (birth year: 2008 or later) Bundesliga, which is the highest division in this age category. Prior to the match day, a preliminary trial was conducted on one of the previous training days in the same sports hall where the team's home match was being played. This trial involved the execution of CMJs and a subjective fatigue and recovery assessment (PRSS, RPE), with the objective of familiarizing the team with the procedures that would be utilized on match day. The corresponding game took place approximately in the middle of the season, i.e., amidst the competitive period. Importantly, only the home team was tracked and assessed. The Kinexon system had already been set up in the sports hall (see description further down). On the day of the match, the players adhered to their usual routines, with the exception that they wore the sports bra, which included an LPS sensor, under their shirts from the warm-up onwards. The tests were conducted by three academically trained sports scientists who were present to provide ongoing guidance to the players and monitored the execution/assessments at all times. Following a brief warm-up period, the players proceeded to perform the LRT and completed the two scales PRSS and RPE prior to continuing the warm-up (pre-game assessment: T_0_). The coach and assistant coach also completed the ROE for each individual player shortly after the beginning of the warm-up (at the same time as the players), i.e., the coach's subjective assessment of the individual players’ fatigue (Figure 1). The same tests were carried out immediately at the start of half-time break by the players (i.e., LRT, PRSS, RPE) and the coaches (ROE) for each individual player (in-game assessment: T_1_). This procedure was then repeated immediately after the game by the players again (LRT, PRSS, RPE) and the coaches for each player (ROE) (post-game assessment: T_2_).

### 2.2 Participants

Eleven adolescent male handball players participated in this study (age: 16.2 ± 0.4 years; body height: 183.5 ± 9.0 cm; body mass: 79.2 ± 11.6 kg) (Table 1). According to the classification method outlined by McKay et al. (24), all participants can be classified as highly trained handball players (Tier level 3), engaging in at least four training sessions weekly, in addition to individual strength and conditioning training, and participating in one match per week. The assessed team is part of the first handball youth league (Bundesliga) in Germany for male b-juniors, which is a regional league encompassing approximately North Rhine-Westphalia with the best teams being promoted to the German championship. The coach of the team reported no injuries, and no players were absent or suffered from any significant physical limitations in the two weeks prior to the match. Written informed consent was provided by parents or legal guardians, and assent was also provided by the participants. The study protocol complied with both the University of Konstanz's ethics guidelines and the Declaration of Helsinki for human testing, and it had been approved by the University of Konstanz's ethics commission (reference number: IRB24KN011-02w).

**Table 1:** Participant characteristics.

Variable	CC	ITT	CC–LOO
Sample size (*n*)	8	11	7
Age (years)	16.1 ± 0.4	16.2 ± 0.4	16.0 ± 0.4
Training experience (years)	8.4 ± 2.4	8.6 ± 2.7	8.6 ± 2.6
Body height (cm)	182.5 ± 6.8	183.5 ± 9.0	182.7 ± 7.3
Body mass (kg)	78.3 ± 11.9	79.2 ± 11.6	79.0 ± 12.8

Data are group mean values ± standard deviations. CC, complete cases (i.e., players that played in both halves); CC-LOO, complete cases–leave-one-out (i.e., players that played ≤ 1 min); ITT, intention-to-treat (i.e., all measured players). External load measures were divided volume-based (i.e., means all measured players).

### 2.3 Assessment of external load parameters by Kinexon

External load parameters were assessed using a local positioning system (Kinexon, version 3.2.6, Munich, Germany; frequency: 16.6 Hz). Volume-based parameters included individual playing time and total distance covered. Although handball matches consist of 60 min of net playing time, referees may stop the clock at their discretion; therefore, Kinexon recordings were paused only at half-time, allowing individual playing times to slightly exceed 60 min.

Intensity-based parameters comprised the number of accelerations and decelerations exceeding ±2.78 m·s^−2^, irrespective of distance. Fast running distance was calculated by aggregating running speed zones of 4.00–5.40 m·s^−1^ (running), 5.41–7.00 m·s^−1^ (high-intensity running), and >7.00 m·s^−1^ (sprinting) into a single metric (25). In addition, metabolic power was derived to estimate the energetic cost of accelerated, decelerated, and constant-speed running. This approach is based on the assumption that accelerated running on flat terrain is energetically equivalent to constant-speed running uphill, as described by Di Prampero et al. (26, 27) and further developed by Di Prampero and Osgnach (28). Instantaneous energy expenditure was estimated from speed and acceleration time-series data, an approach proposed to better capture the energetic demands of intermittent team sports compared with speed- or acceleration-based metrics alone (28, 76).

Players wore a lightweight inertial measurement unit embedded in a sports bra positioned between the scapulae (11). Devices transmitted ultra-wideband signals to external receivers throughout the match, with data processed on a central computer (12). The system was installed, calibrated, and accuracy-checked by a sport scientist following standardized procedures for competitive handball environments. Nine antennas were positioned around the court and connected to anchor antennas installed at three different heights within the arena (76). Player positions were calculated using time-of-flight measurements of ultra-wideband signals (25), smoothed via an unscented Kalman filter, and processed using triangulation methods. Speed and acceleration data were derived from positional differentiation and filtered using zero-phase low-pass Butterworth filters [cut-off frequencies: 1.0 Hz for speed and 0.5 Hz for acceleration (76)]. The validity of the Kinexon system for handball has been demonstrated previously (10, 13).

Recording commenced with the referees’ initial whistle and was paused at half-time (30 min). The same procedure was applied during the second half, with recording terminated immediately after the final whistle (13).

### 2.4 Leg recovery test

Internal objective fatigue was assessed using the LRT, a CMJ–based measure of lower-limb neuromuscular function. Following the initial phase of the standardized warm-up, which included light running and mobility exercises, each participant performed three CMJs. Jump performance was assessed using a Polar Vantage V2 sports watch, in which the LRT is implemented. Prior to the study, a pilot testing phase was conducted during one of the training sessions to ensure that all participants were familiar with the demands of the test. The CMJs were performed in accordance with the procedures and descriptions outlined by Bosco et al. (29). During the execution of the jumps, participants were required to position their hands on their hips while maintaining an upright posture with their legs nearly straight. Vibration and sound signals were generated by the watch prior to each of the three jumps. The participants were required to commence the execution by squatting as rapidly as possible, with the knees bent to approximately 90°. This was followed by a simultaneous, maximal dynamic straightening of both legs, with the objective of jumping as high as possible. During the flight phase, the legs were required to be held straight (knee angle: 180°) and they were only permitted to be bent immediately prior to touchdown to ensure a gentle landing. Holding the arms akimbo was required throughout the entirety of the jumps. The inertial measuring unit integrated in the wristwatch (Polar Vantage V2) calculated the mean jump height (cm) of all three jumps (corresponds to the LRT score). The mean jump height of repetitive trials is recommended, as research analysing CMJ measurements demonstrated that the average jump height of CMJs provides more reliable information regarding an athlete's neuromuscular status compared to the highest jump (30).

### 2.5 Assessment of perceived exertion and recovery by the players

Two different single-item questionnaires were utilized in the study. The first was the RPE scale, which is based on the principle that athletes can monitor their physiological strain during exercise and retrospectively assess perceived fatigue (31, 32). This scale has been widely applied in contemporary monitoring research, including recent work highlighting its relevance for assessing training and match demands (33). The version of the RPE scale that was utilized was the 6–20 scale, where 6 indicates no perceived exertion and 20 indicates the highest possible exertion. The second tool that was employed was the PRSS (34), which also considers life load factors (diet, sleep habits, etc.) and recovery times. The PRSS is a psychobiological tool that is utilized for the identification of the state of recovery following different kinds of loads, with a scale ranging from 0 to 10 (where 0 denotes a state of very poor recovery/extreme fatigue, and 10 denotes a state of very good recovery/high energy levels). Beginning with the warm-up of the match, all subjects were requested to rate their perceived level of recovery (PRSS) and RPE. This rating was repeated at the beginning of the half-time break (i.e., after the first 30 min. of the game) and once more immediately after the match (post-game; after 60 min. of the game) (Figure 1).

### 2.6 Assessment of perceived exertion of the players as rated by the coaches

Coaches rated players’ observed exertion using the same 6–20 Borg scale as players’ RPE, thereby aligning ROE with the established RPE methodology. Coaches’ subjective assessments of players’ fatigue (ROE) were collected at three time points: before the match (T_0_), at half-time immediately after the first half (T_1_), and after the second half (T_2_). A visual analogue scale ranging from 6 (no fatigue) to 20 (highest possible fatigue) was provided to both coaches (head coach and assistant coach), who rated each of the 14 players irrespective of individual playing time. Coaches’ ROEs were intended to capture overall match-related exertion and therefore inherently reflected differences in individual playing time. These ratings are hereafter referred to as ROE1 (head coach) and ROE2 (assistant coach). The head coach holds the highest coaching license level in Germany, and the assistant coach holds the second-highest level; both have more than ten years of experience in youth handball and have worked with most players for at least two years. No formal *a priori* inter-rater calibration was performed, as ROE was conceptualized as an exploratory observer-based measure. However, prior to data collection, coaches were familiarized with the ROE scale and the assessment procedure, and agreement between coaches was examined a posteriori.

### 2.7 Statistical analyses

Our primary analysis set comprised *complete cases* (CC), defined as players with usable measurements for both half and end of the game and all variables required for a given contrast. Robustness was examined in sensitivity analyses using *CC–leave-one-out* (CCLOO) excluding players with conspicuously low playing time and an *intention-to-treat* (ITT) set including all measured players. External load measures were divided volume-based (i.e., individual playing time, TDC) and intensity-based (i.e., accelerations; decelerations; fast running; metabolic power) parameters. Internal load measures were separated into objective (i.e., LRT) and subjective (i.e., RPE, ROE, PRSS) parameters.

Given the small sample size and uncertainty regarding normality assumptions, both Pearson's *r* and Spearman's *ρ* were calculated to examine the robustness of observed associations. Associations between external and internal load measures were examined descriptively in JASP (version 0.19.2), with correlation coefficients reported alongside two-sided *p*-values and 95% confidence intervals (CI). Values were interpreted as weak (*r* = 0.10–0.35), moderate (*r* = 0.36–0.67), or strong (*r* = 0.68–1.00) (35). Correlations were stratified by category: external load measures divided by volume-based and intensity-based parameters compared to internal load measures divided by objective and subjective parameters and were treated as exploratory without multiplicity adjustment at *α* = 0.05. Sensitivity analyses were conducted within each analysis set as described above. Importantly, the subjective measurements after half of the game were correlated with the accumulated or averaged parameters over the whole game.

Agreement between the coaches’ ratings and players’-reported fatigue was quantified with Lin's concordance correlation coefficient (*CCC*) and 95% CI, which jointly reflect accuracy and precision of absolute agreement (36). We additionally reported Bland–Altman mean bias and 95% limits of agreement (LoA) for method comparison (37). As complementary reliability indices, we computed intra-class correlation coefficient *ICC*_(3,1)_ and *ICC*_(3,k)_ from the two-way mixed, consistency model, following the Shrout–Fleiss and McGraw–Wong conventions and reporting guidance by Koo and Li (38). Values were interpreted as poor (*ICC*_(3,k)_ < 0.50), moderate (*ICC*_(3,k)_ between 0.50 and <0.75), good (*ICC*_(3,k)_ between 0.75 and <0.90), and excellent (*ICC*_(3,k)_ ≥ 0.90).

A formal decision on agreement used Shieh's exact test for limits-of-agreement equivalence. All agreement procedures were implemented in Jamovi with the SimplyAgree package (39) using the prespecified simple (non-nested) model for the primary analyses, while nested/replicate options were used only in sensitivity checks.

Given the exploratory study design, Bland–Altman plots were used primarily for descriptive and illustrative purposes rather than to establish precise limits of agreement.

## 3 Results

### 3.1 Descriptive statistics

Participants included eight players in the *CC* primary set, eleven players in the *ITT* set, and seven players in the *CC–leave-one-out set* (Table 1). Table 2 displays the descriptive statistics (*M* ± *SD*) by category and subcategory for the first and second half of the game as well as the whole game. In terms of external load measures, volume-based (i.e., individual playing time, TDC) as well as the intensity-based (i.e., accelerations, decelerations, fast running, and metabolic power) parameters were greater in the first half compared to the second half of the game. Regarding internal load measures, the LRT height (objective parameter) was greater in the first than the second half of the game. Conversely, the subjective parameters RPE and ROE increased and PRSS decreased from the first to the second half of the game.

**Table 2:** Descriptive statistics for the complete cases (*n* = 8) by category and subcategory for the pre-game testing, the testing at end of the first and second half of the game, and the whole game.

Category	Subcategory	Variable	Pre-game	1st half	2nd half	Total
External load measures	Volume-based parameters	Individual playing time (min)	*N/A*	25.75 ± 8.01	24.62 ± 9.49	50.4 ± 17.4
		TDC (m)	*N/A*	2,064.1 ± 820.8	1,785.5 ± 703.2	3,849.6 ± 1,510.6
	Intensity-based parameters	Accelerations (n)	*N/A*	4.12 ± 3.04	4.00 ± 5.10	8.12 ± 7.72
		Decelerations (n)	*N/A*	1.38 ± 1.30	1.38 ± 1.77	2.75 ± 2.96
		Fast running (m)	*N/A*	814.0 ± 521.8	637.4 ± 411.3	1,451.4 ± 927.9
		Metabolic power (W·kg^−1^)	*N/A*	4.91 ± 1.07	4.68 ± 1.18	4.80 ± 1.09
Internal load measures	Objective parameter	LRT (cm)	36.62 ± 3.34	35.88 ± 3.94	35.75 ± 4.80	*N/A*
	Subjective parameters	PRSS (0–10)	8.25 ± 1.91	6.12 ± 1.89	4.75 ± 2.43	*N/A*
		RPE (6–20)	8.00 ± 2.45	14.62 ± 2.20	16.12 ± 2.85	*N/A*
		ROE1 (6–20)	11.00 ± 1.51	15.38 ± 3.07	17.00 ± 2.00	*N/A*
		ROE2 (6–20)	6.62 ± 0.74	12.38 ± 1.85	15.50 ± 1.31	*N/A*

Data are group mean values ± standard deviations. LRT, leg recovery test; N/A, not available; PRSS, Perceived Recovery Status Scale; ROE, coaches’ rating of observed exertion (1 = head coach, 2 = assistant coach); RPE, players’ rating of perceived exertion; TDC, total distance covered.

### 3.2 Associations between external and internal load measures

Associations between external (i.e., volume-based and intensity-based parameters) and internal (objective and subjective parameters) load measures are displayed in Table 3. *After the end of the first half*, the analyses revealed non-significant moderate magnitude positive correlations between the internal load objective measure LRT and the external volume-based load measures individual playing time (*r* = 0.513) and TDC (*r* = 0.493). Significant moderate-to-strong correlations were observed between the subjective internal load measures and the volume-based external load indicators individual playing time and TDC (PRSS: *r* = −0.661 and *r* = −0.695, both *p* < .05; RPE: *r* = 0.766 and *r* = 0.782, both *p* < .01). In terms of external intensity-based load measures, we observed non-significant weak to moderate positive correlations with LRT (accelerations: *r* = 0.396, decelerations: *r* = 0.100, fast running: *r* = 0.520, metabolic power: *r* = 0.188). Moreover, partially significant moderate to strong magnitude negative and positive correlations were detected between the internal load subjective measures and the external intensity-based load measures accelerations (PRSS: *r* = −0.461; RPE: *r* = 0.556), decelerations (PRSS: *r* = −0.665, *p* < .05; RPE: *r* = 0.630, *p* < .05), fast running (PRSS: *r* = −0.514; RPE: *r* = 0.615, *p* < .05), and metabolic power (PRSS: *r* = −0.477; RPE: *r* = 0.812, *p* < .01), again indicating that recovery decreased and exertion increased when the intensity-based parameters increased.

**Table 3:** Pearson's correlation coefficients between external and internal load measures after the end of the first half and second half of the game.

Variables	LRT	PRSS	RPE
End of the 1st half			
Individual playing time	.513	-.661*	.766**
TDC	.493	-.695*	.782**
Accelerations	.396	-.461	.566
Decelerations	.100	-.665*	.630*
Fast running	.520	-.514	.615*
Metabolic power	.188	-.477	.812**
End of the 2nd half			
Individual playing time	.529	-.679*	.875**
TDC	.512	-.676*	.897**
Accelerations	.537	.072	.363
Decelerations	.194	-.286	.463
Fast running	.527	-.390	.698*
Metabolic power	.628*	-.302	.637*

LRT, leg recovery test; PRSS, Perceived Recovery Status Scale; RPE, players’ rating of perceived exertion; TDC, total distance covered.

* Indicates statistically significant correlation at *p* < .05.

** Indicates statistically significant correlation at *p* < .01.

*After the end of the second half*, the analyses revealed non-significant moderate magnitude positive correlations between the internal load objective measure LRT and the external volume-based load measures individual playing time (*r* = 0.529) and TDC (*r* = 0.512). In addition, significant moderate-to-strong correlations were observed between subjective internal load measures and volume-based external load indicators. PRSS was negatively associated with individual playing time (*r* = −0.679, *p* < .05) and TDC (*r* = −0.676, *p* < .05), whereas RPE showed strong positive associations with both playing time (*r* = 0.875, *p* < .01) and TDC (*r* = 0.897, *p* < .01). These findings indicate that perceived recovery decreased, while perceived exertion increased, with greater playing time and total distance covered. Regarding intensity-based external load measures, weak to moderate positive associations with LRT performance were observed (accelerations: *r* = 0.537; decelerations: *r* = 0.194; fast running: *r* = 0.527; metabolic power: *r* = 0.628). However, none of these associations reached statistical significance. Lastly, partially significant weak to strong magnitude negative and positive correlations were detected between the internal load subjective measures and the external intensity-based load measures accelerations (PRSS: *r* = 0.072; RPE: *r* = 0.363), decelerations (PRSS: *r* = −0.286; RPE: *r* = 0.463), fast running (PRSS: *r* = −0.390; RPE: *r* = 0.698, *p* < .05), and metabolic power (PRSS: *r* = −0.302; RPE: *r* = 0.637, *p* < .05), again indicating that recovery decreased and exertion increased when the intensity-based parameters increased.

### 3.3 Agreement between the coaches’ ratings and players’-reported fatigue

**Figure 2:**
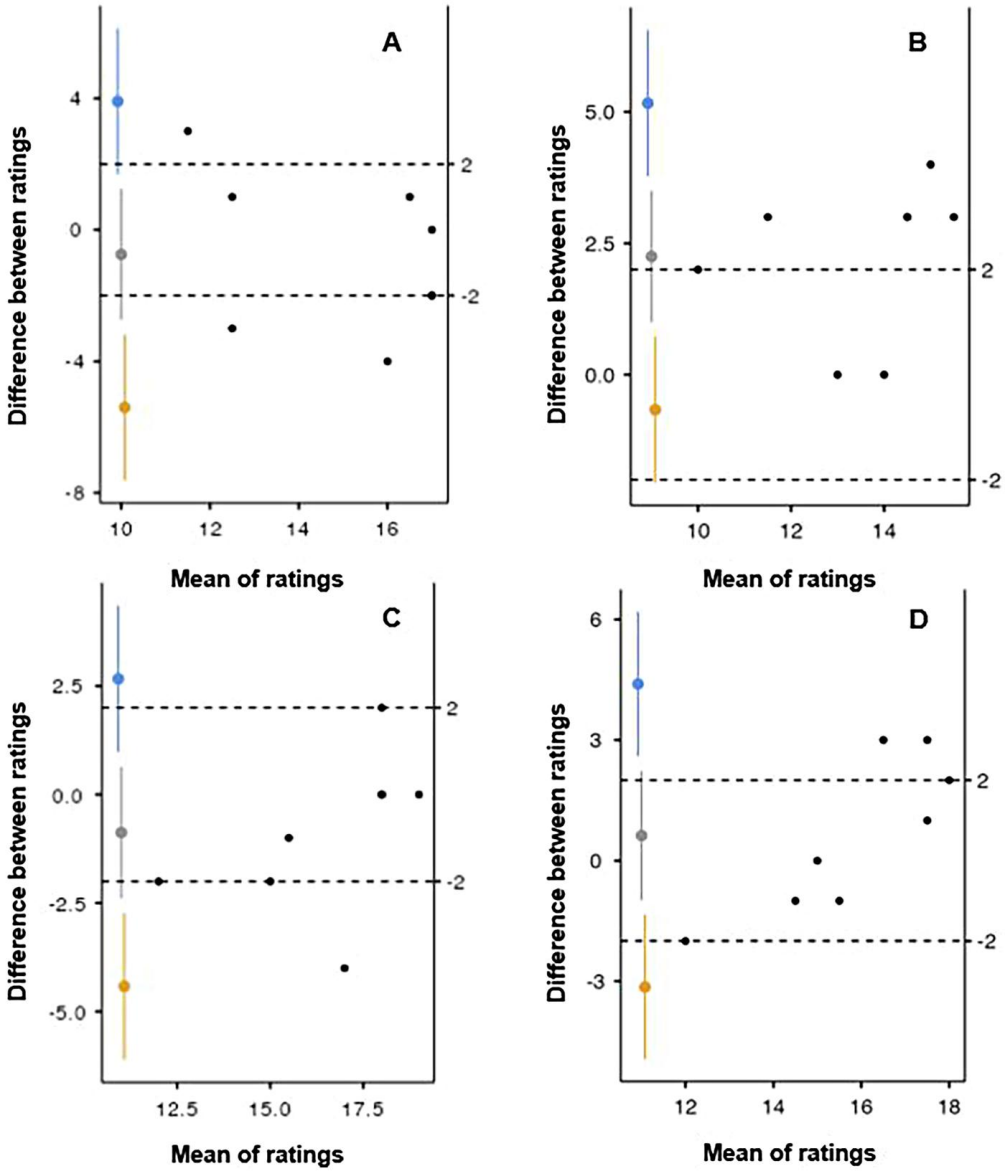
Bland–Altman plots for the comparison of the players’ rating of perceived exertion (RPE) with the coaches’ rating of observed exertion (ROE) after the first half [**(A)** players’ RPE vs. head coach ROE, **(B)** players’ RPE vs. assistant coach ROE] and the second half [**(C)** players’ RPE vs. head coach ROE, **(D)** players’ RPE vs. assistant coach ROE] of the game. The 95% confidence intervals are indicated by the dashed lines.

The primary agreement analysis between the coaches’ ratings and players’-reported fatigue is shown in Table 4 and illustrated in Figures 2A–D. After the end of the first half of the game, concordance between players’ RPE and the head coach's rating (ROE1) was good (*CCC* = 0.58, *ICC*_(3,k)_ = 0.76) with a negative mean bias for RPE–ROE1 of −0.75, indicating that ROE1 tended to rate higher than players (Figure 2A). Concordance between RPE and the assistant coach's rating (ROE2) was also good (*CCC* = 0.43; *ICC*_(3,k)_ = 0.85) with a positive mean bias for RPE–ROE2 of 2.25, indicating that ROE2 tended to rate lower than players (Figure 2B). After the end of the second half of the game, concordance between players’ RPE and the head coach's rating (ROE1) increased and was good (*CCC* = 0.68, *ICC*_(3,k)_ = 0.84) with a negative mean bias for RPE–ROE1 of −0.88, still indicating that ROE1 tended to rate higher than players (Figure 2C). Concordance between RPE and the assistant coach's rating (ROE2) decreased and was good (*CCC* = 0.60; *ICC*_(3,k)_ = 0.77) with a positive mean bias for RPE–ROE2 of 0.63, still indicating that ROE2 tended to rate lower than players (Figure 2D).

**Table 4:** Results of the agreement analyses (i.e., complete cases–leave-one-out; *n* = 7).

Comparison	CCC	ICC_(3,k)_	Lower LoA	Mean bias	Upper LoA
1st half					
RPE *vs.* ROE1	0.58 [−0.05; 0.88]	0.76 [0.09; 0.94]	−5.41 [−7.62; −3.19]	−0.75 [−2.74; 1.24]	3.91 [1.69; 6.12]
RPE *vs.* ROE2	0.43 [−0.01; 0.73]	0.85 [0.41; 0.96]	−0.67 [−2.05; 0.72]	2.25 [1.01; 3.94]	6.17 [3.78; 6.56]
2nd half					
RPE *vs.* ROE1	0.68 [0.16; 0.91]	0.84 [0.41; 0.96]	−4.42 [−6.11; −2.73]	−0.88 [−2.39; 0.64]	2.67 [0.98; 4.36]
RPE *vs.* ROE2	0.60 [0.23; 0.82]	0.77 [0.14; 0.94]	−3.14 [−4.94; −1.35]	0.63 [−0.98; 2.23]	4.39 [2.60; 6.19]

Data are concordance correlation coefficient (*CCC*), intra-class correlation coefficient (*ICC*), and 95% limits of agreement (LoA). LRT, leg recovery test; N/A, not available; ROE, coaches’ rating of observed exertion (1 = head coach, 2 = assistant coach); RPE, players’ rating of perceived exertion.

## 4 Discussion

The primary finding of the present study is that subjective fatigue measures, particularly RPE and PRSS, were more responsive to the cumulative demands of match play than the applied objective neuromuscular assessment, highlighting clear differences between subjective and objective approaches to fatigue monitoring under competitive match conditions.

Rather than reflecting isolated physical capacities, these subjective measures likely capture an integrative response to match exposure, encompassing physiological strain, cognitive demands, and emotional stressors. This interpretation is consistent with previous work showing that subjective monitoring tools may outperform objective neuromuscular markers when assessing short-term fatigue and situational stress in applied sport settings (19, 40). Taken together, these findings align with contemporary frameworks conceptualizing fatigue as a multidimensional psychophysiological response shaped by the interaction of external demands, internal perception, and contextual factors.

Given the exploratory and single-match design, the following interpretations should be considered preliminary and primarily serve to inform future, larger-scale investigations.

The findings indicate that coaches’ ratings of observed exertion showed increasing agreement with players’ perceived exertion as match exposure progressed. This convergence likely reflects the greater visibility of fatigue-related behaviours and effort cues under accumulated load and aligns with findings from other invasion sports, where coach–athlete agreement improves as physical and contextual demands intensify (20, 41, 42). From an applied perspective, this growing alignment reinforces the value of coach-based observational ratings as a practical complement to athlete self-reports, particularly in dynamic team sports such as handball, where substitution and rotation decisions must be made in real time.

In contrast, the absence of a clear response in the objective performance-based fatigue indicator (LRT) suggests that neuromuscular performance may be maintained during highly intermittent match play, even when athletes report increasing levels of fatigue. Such dissociation between perceptual and performance-based fatigue has been reported previously and may reflect compensatory strategies, pacing behaviour, or the multifactorial nature of fatigue during competition, where central and perceptual components play a prominent role alongside peripheral mechanisms (43, 44).

### 4.1 Relationship between external and internal (objective) load measures

The present findings indicate that the objective neuromuscular component, assessed via the LRT, showed no systematic association with external load exposure across the match. Rather than reflecting a dose–response relationship between accumulated match demands and neuromuscular fatigue, LRT scores remained largely stable across measurement time points. This suggests that, under competitive handball conditions, neuromuscular performance assessed via isolated explosive tasks may be relatively preserved despite substantial external load exposure.

When situating these findings within the existing literature, it is important to distinguish between methodological approaches. Most previous handball studies have employed pre–post designs and reported absolute reductions in CMJ performance or comparable neuromuscular markers following competitive matches, simulated games, or tournament play [e.g., (45, 46)]. Such studies provide valuable insight into the general magnitude of match-induced fatigue but differ conceptually from the present dose–response approach, which focused on whether higher individual external loads were associated with greater neuromuscular performance decrements during the match. Consequently, earlier pre–post findings are not directly comparable, as they describe overall fatigue responses rather than determinants of within-match performance variation.

The observed dissociation between external workload and neuromuscular performance aligns with conceptual models emphasizing that internal load does not necessarily scale linearly with external load, as individual physiological, perceptual, and contextual factors can decouple the two domains (44).

Importantly, the absence of strong associations between external load exposure and LRT performance should not be interpreted as a lack of relevance of neuromuscular testing *per se*. Rather, the LRT appears to capture a specific dimension of fatigue related to anaerobic neuromuscular performance capacity. From a practical perspective, this dimension is highly relevant in handball, where decisive actions frequently occur under pronounced subjective fatigue. In such situations, the key question is not only whether an athlete feels fatigued, but whether they can still produce short-term, high-intensity actions comparable to earlier phases of the match.

The present findings therefore suggest that the LRT reflects a performance-oriented fatigue component that is only partially aligned with athletes’ subjective perceptions of exertion or recovery. While this neuromuscular dimension seems to play a subordinate role in subjective fatigue regulation during match play, it may nonetheless be critical for evaluating an athlete's capacity to execute decisive explosive actions under fatigue. Consequently, neuromuscular tests such as the LRT should be viewed as complementary tools within a multidimensional fatigue monitoring framework rather than as direct substitutes for subjective fatigue measures.

Studies in adult handball players have reported post-match reductions in CMJ performance following competitive or simulated match play (45, 46), as well as during tournament settings characterized by substantially higher cumulative workloads (47). Comparable decrements have also been observed in adolescent players following multi-match tournament exposure (48). In contrast, the present findings are consistent with Bauer et al. (40), who reported stable LRT performance after a single training session in adolescent players and attributed this to effective recovery opportunities inherent to handball's intermittent structure. Similar interpretations have been proposed in broader reviews of handball, highlighting position-specific demands and embedded recovery phases during match play (49).

Physiological considerations further support this interpretation. Mechanisms underlying transient neuromuscular fatigue—such as altered membrane excitability due to K^+^ accumulation, Na^+^/K^+^-pump regulation, or metabolic perturbations involving phosphocreatine depletion and metabolite accumulation—are known to recover rapidly during intermittent exercise patterns (50–52). Consistent with this, biochemical data from handball indicate relatively low post-match lactate concentrations, reflecting a predominantly aerobic metabolic profile that may facilitate rapid recovery of explosive performance (46, 53, 54). Contemporary insights into lactate metabolism further support the notion that efficient clearance and metabolic recovery can attenuate short-term neuromuscular impairment (55).

Overall, the absence of meaningful associations between LRT performance and external load suggests that the LRT may have limited sensitivity for detecting the rapidly fluctuating fatigue patterns characteristic of handball match play. Nevertheless, its ecological relevance remains intact, as players are often able to execute isolated explosive actions consistently across a match, even when subjective fatigue accumulates. This interpretation aligns with broader frameworks conceptualizing internal load as a multidimensional construct shaped by physiological, psychological, and contextual influences rather than by external mechanical work alone (44).

### 4.2 Relationships between external load measures and internal (subjective) load measures

The present findings indicate that RPE is closely linked to match-related external load exposure and appears sensitive to both volume- and intensity-related demands. Rather than reflecting a single aspect of load, perceived exertion seems to integrate cumulative playing time, locomotor volume, and intensity components of match play. This supports the notion that RPE functions as a global indicator of match stress rather than a proxy for isolated physical parameters. Given the limited number of match-based studies in handball—which have primarily focused on describing external match demands or pre–post fatigue responses rather than dose–response relationships [e.g., (46, 49, 56)]—training studies provide an important comparative framework. Across different populations and settings, previous research has consistently demonstrated meaningful associations between RPE and external load metrics such as total distance covered or composite load measures (57–59). These observations are further supported by the broader synthesis provided by McLaren et al. (5), who identified total distance covered as the external load variable most consistently related to RPE across team sports. Evidence from competitive soccer similarly suggests that the relationship between RPE and external load persists across training and match contexts, albeit with varying strength depending on situational demands (60).

The strong associations observed between RPE and multiple external load indicators in the present study likely reflect the multidimensional nature of perceived exertion. RPE represents an integrative psychophysiological response encompassing metabolic strain, neuromuscular effort, cognitive load, and emotional factors. In invasion games such as handball, frequent decision-making, perceptual demands, and tactical problem solving contribute substantially to overall exertion (61). These contextual and cognitive components are not directly captured by LPS- or GPS-derived external load measures but are inherently reflected in players’ subjective perception of effort. Consequently, RPE may offer value for monitoring match-induced fatigue in complex, high-decision environments such as competitive handball.

Associations with volume-based external load measures suggest that greater playing time and higher locomotor demands are linked to lower perceived recovery. Compared with RPE, however, these relationships were generally weaker, supporting the notion that PRSS captures a distinct, more recovery-oriented dimension of internal load rather than momentary exertion.

From a conceptual perspective, PRSS reflects not only acute match demands but also broader recovery-related influences, including sleep, nutrition, and between-session recovery status (34). This broader psychobiological scope may explain its sensitivity to accumulated strain rather than immediate physical effort. Evidence from elite female handball further supports this interpretation, demonstrating pronounced positional and role-specific variation in subjective well-being and internal load responses during competition (62). Together, these findings highlight the importance of individualized recovery monitoring in team sports.

The timing of PRSS assessment provides additional context for interpretation. As measurements were obtained at the beginning of the half-time break, the observed decline reflects players’ recovery state immediately following the first half rather than the effects of the subsequent rest period. This distinction is relevant, as recovery-related perceptions may evolve rapidly during short breaks. Comparable findings from elite handball indicate that psychometric recovery markers are particularly sensitive to cumulative load exposure, especially during periods of increased competitive density (63).

Only limited research has examined PRSS responses around match play. In youth football, Paul et al. (64) reported marked post-match reductions in perceived recovery, although differences between players with varying playing time were not evident. Methodological differences, including dichotomous grouping of playing time, may have limited sensitivity to load-dependent variation. In contrast, the continuous analytical approach applied in the present study allows a more nuanced examination of the relationship between match exposure and perceived recovery.

The dissociation between declining PRSS scores and the absence of corresponding changes in neuromuscular performance further suggests that subjective recovery markers may detect early or low-level fatigue states that are not captured by performance-based tests (43, 65–67). Such dissociation is consistent with evidence indicating that athletes can often maintain short-duration explosive performance despite increasing subjective fatigue, particularly in intermittent sports. Previous research in youth handball similarly highlights the superior sensitivity of psychometric indicators compared with objective physiological measures when assessing readiness and fatigue-related changes (68). In this context, the substantial cognitive and perceptual demands of handball (61) may further contribute to discrepancies between subjective recovery perceptions and isolated neuromuscular performance outcomes.

### 4.3 Relationship between the subjective assessments of the players’ fatigue by the coaches and those of the individual players

The present findings demonstrate a progressively increasing alignment between players’ perceived exertion and coaches’ observational ratings across the match. This convergence suggests that coaches’ assessments become increasingly reflective of players’ internal fatigue states as match exposure accumulates. Such agreement is consistent with meta-analytic evidence indicating moderate-to-high correspondence between perceived internal load and coach-derived load indices across team sports (23), as well as with a systematic review reporting comparable relationships between athletes’ RPE and coaches’ ROE, particularly under conditions of higher load (20). Previous research from team sports with structural similarities to handball provides further context for these findings. Studies in youth soccer and elite invasion sports have consistently reported meaningful associations between athlete- and coach-derived exertion ratings (21, 41, 69). Importantly, observational fatigue assessment has been shown to rely on a diverse set of perceptual cues, including movement quality, external signs of fatigue, breathing patterns, running style, and overall intensity (21). This highlights the inherently multifactorial nature of coach-based exertion ratings and underscores their sensitivity to externally observable fatigue-related behaviours rather than isolated physiological states. The increasing agreement between RPE and ROE over the course of the match may be explained by the expanding observational window available to coaches. Early assessments are largely informed by brief warm-up impressions, whereas prolonged match exposure enables coaches to integrate a broader range of behavioural, technical, and movement-related cues. In contrast, players` perceived exertion incorporates additional internal and contextual factors—such as sleep quality, hydration status, academic stress, and nutrition—that remain largely inaccessible to external observers (70). These differing information sources may account for greater discrepancies early in the match and increasing convergence as observable fatigue cues become more pronounced. Taken together, the present findings suggest that coaches can provide increasingly accurate real-time assessments of player fatigue during competition. In the context of handball, where timely substitution and rotation decisions are integral to performance management, coach-based observational ratings appear to represent a practical and ecologically valid complement to athlete self-reports, particularly as match duration and cumulative load increase.

### 4.4 Implications

From a practical perspective, the findings suggest that subjective measures such as RPE and PRSS may be more valuable for monitoring in-game load in adolescent handball players than objective neuromuscular testing. The following practical implications should be interpreted with caution. Given the exploratory and preliminary nature of the study, the small number of complete cases, and the single-match design, these implications are intended to provide initial, hypothesis-generating guidance rather than definitive recommendations. In this context, coaches are required to manage not only substitutions but also the rhythm, pace, and interruption structure of the match, all of which may influence players’ perceived exertion and recovery. Unofficial and official game stoppages, such as wiping time-outs or referee-dependent delays, vary substantially in frequency and duration and can temporarily influence players’ fatigue states (71). Coaches must therefore assess fatigue dynamically rather than relying on static load indicators. High levels of fatigue may at times be accepted for tactical reasons when key players remain essential to the game plan. Thus, although no group-level associations were observed between external load and objective internal load (LRT), individual interactions may still be relevant: players who demonstrate high external but low subjective internal load may represent “rapid adapters” who recover quickly and tolerate high workloads (4).

The subjective measures applied in the present study—RPE and PRSS—capture partially distinct aspects of fatigue. RPE primarily reflects acute exertion, whereas PRSS is more sensitive to recovery status following prior workloads (34). Their negative association observed in the present study is consistent with their conceptual foundations, indicating that perceived exertion increases as perceived recovery declines. For in-game or immediate post-game assessment, the present findings suggest that RPE alone may provide a sufficiently sensitive indicator of acute fatigue. In contrast, the limited sensitivity of the CMJ-based LRT for detecting immediate post-match fatigue in handball may be related to the intermittent nature of the sport, in which fatigue does not necessarily peak immediately after workload exposure (40, 56, 72). Given that jump-based tests often show limited responsiveness to very acute post-exercise fatigue (73), but are widely recommended for monitoring neuromuscular status over short-, mid-, and long-term periods (30), such tests may be more suitable for tracking fatigue trends beyond the immediate post-match window rather than for in-game decision-making.

The increasing agreement between players’ RPE and coaches’ ROE across the match carries additional practical relevance. Subjective internal load assessments facilitate both conscious pacing adjustments by players and informed situational substitutions by coaching staff. Such decisions are difficult to justify using external load metrics alone—such as total distance covered—which do not capture perceptual, psychological, or cognitive components of fatigue (74). The internal and external loads accumulated during a match further influence subsequent training requirements. When match load is insufficient, additional top-up conditioning, i.e., supplementary training stimuli, may be required to ensure that players achieve the intended weekly stimulus. Conversely, when match demands induce substantial neuromuscular or perceptual fatigue, tapering strategies—such as reduced volume, modified intensity, or a technical–tactical emphasis—may be necessary to facilitate recovery and protect performance capacity. Accordingly, external, subjective, and objective load indicators should be integrated within a multidimensional decision-support framework rather than interpreted in isolation. Despite the exploratory and single-match nature of the study, integrating load data within tactical demands, substitution possibilities, and competition schedules may support more informed, athlete-centred coaching decisions in competitive handball (75).

### 4.5 Limitations

First, the study adopted an ecologically valid in-game design based on a single official match. As a result, the sample size was determined by the structural constraints of competitive handball match play, and no *a priori* sample size calculation was conducted, since the study was exploratory in nature and not intended for confirmatory inference. As data were derived from a single competitive match, the present findings do not account for between-match variability and should therefore be interpreted as context-specific. Given the small sample size, agreement and equivalence analyses were interpreted in an exploratory and descriptive manner. In particular, Bland–Altman plots were used to visually illustrate agreement rather than to establish precise limits of agreement, and Shieh's equivalence testing was not used to draw confirmatory conclusions regarding statistical equivalence. Wide confidence intervals for several correlations further indicate uncertainty in effect size estimation. Second, data were collected from a single adolescent male team, which limits the generalizability of the findings to other age groups, genders, or competitive levels. Moreover, different tactical approaches—such as more offensive or defensive playing styles—may substantially influence both external load indicators and fatigue development, limiting the transferability of the present findings to teams with different tactical profiles. Third, the assessment of fatigue at only three discrete time points (pre-game, half-time, post-game) does not capture the continuous and fluctuating nature of fatigue in handball and may have overlooked short-term peaks during high-intensity phases. Fourth, the study focused exclusively on metabolically derived external load variables from the LPS system; mechanical load components such as body contacts, jumps, or collisions—highly relevant in handball—were not quantified. Fifth, previous training load, accumulated fatigue, and potential match congestion were not monitored, although these factors may influence internal load responses. Finally, because only one team and two coaches were analysed, positional differences and between-coach variability could not be examined. Accordingly, correlation and agreement analyses should be interpreted as exploratory rather than confirmatory.

### 4.6 Outlook

Future research should address these limitations by adopting longitudinal, multi-match designs that capture the dynamic nature of fatigue across competitive cycles. Season-long monitoring may help clarify when neuromuscular, perceptual, or tactical fatigue accumulates and how these components interact over time. Expanding load assessment to include mechanical variables—such as impacts, and jumps—would provide a more comprehensive picture of the demands faced by handball players. Finally, examining the stability of ROE–RPE relationships across different teams, coaching styles, and competitive levels could support the development of more individualized and ecologically valid training and match load management strategies.

## 5 Conclusion

This study showed that subjective indicators of fatigue (RPE and PRSS) were more sensitive to match-derived load than peak neuromuscular response measured by the LRT, which remained relatively stable across the match and exhibited only limited associations with external load. Coaches were able to reliably recognize players’ subjective fatigue, with agreement between RPE and ROE increasing as the match progressed. In applied settings where detailed locomotor data are unavailable, individual playing time appears to be a practical proxy for accumulated load; however, replication in other settings and in a larger number of players is needed to validate this observation.

## Data Availability

The raw data supporting the conclusions of this article will be made available by the authors, without undue reservation.
